# Applying Apriori algorithm to explore long-term care services usage status—Variables based on the combination of patients with dementia and their caregivers

**DOI:** 10.3389/fpsyg.2022.1022860

**Published:** 2022-12-13

**Authors:** Yen-Jen Chen, Kai-Ming Jhang, Wen-Fu Wang, Guan-Cheng Lin, Shao-Wei Yen, Hsin-Hung Wu

**Affiliations:** ^1^Department of Psychiatry, Changhua Christian Hospital, Changhua, Taiwan; ^2^Department of Neurology, Changhua Christian Hospital, Changhua, Taiwan; ^3^Department of Recreation and Holistic Wellness, Ming Dao University, Changhua, Taiwan; ^4^Department of Business Administration, National Changhua University of Education, Changhua, Taiwan; ^5^Department of Information Management, National Changhua University of Education, Changhua, Taiwan; ^6^Department of M-Commerce and Multimedia Applications, Asia University, Taichung, Taiwan; ^7^Faculty of Education, State University of Malang, Malang, East Java, Indonesia

**Keywords:** long-term care services, patients with dementia, caregivers, patient–caregiver dyad, Apriori algorithm, home personal care services, assistive devices

## Abstract

**Purpose:**

The aim of this study was to identify the combination of patients with dementia and their caregivers' characteristics associated with long-term care (LTC) services usage.

**Patients and methods:**

A cross-sectional study was conducted with 475 patients with mild, moderate, and severe dementia at Changhua Christian Hospital, Taiwan. Eleven types of variables from patients with dementia, nine types of variables from patients' caregivers, and 15 types of LTC services were used for this study. The Apriori algorithm was employed to identify the attributes from the patients and their caregivers who used a particular LTC service from a comprehensive viewpoint.

**Results:**

A total of 75 rules were generated by the Apriori algorithm with support of 2%, confidence of 80%, and lift >1. Among these rules, 25 rules belonged to home personal care services which were summarized further into four general rules for home personal care services. On the other hand, 50 rules belonged to assistive devices that were summarized further into 21 general rules based on their similarities. Patient's walking ability, patient's emotional liability, unemployed or retired caregivers, caregivers' feelings with either helplessness or hopelessness, and caregivers who cared for patients with dementia solely were found to be the critical variables to use home personal care services. In contrast, patient's walking ability, age, and severity as well as caregivers' age, mood, marital status, caregiving burden, and the patient being cared for mainly by a foreign care helper were found to be the critical variables to use assistive devices.

**Conclusion:**

This study showed preliminary results on the LTC service usage from patients with dementia and their caregivers residing in the community. Understanding the patient–caregiver dyad's profile leads the service providers, policymakers, and the referral team to tailor service provisions better to meet the needs and identify the potential target groups. The findings in this study serve as references to reduce caregivers' burden as well as to improve the quality of care for patients with dementia.

## Introduction

Population aging is one of the major public health issues influencing all developed countries including Taiwan. Taiwan is an aged society in 2022 and is predicted to become a superaged society by 2025. The number of patients with dementia has increased dramatically with rapid growth of the elderly population. In a nation-wide population-based study in Taiwan, the prevalence of dementia increased significantly from 4.7 per 100 people to 7.6 per 100 people from 2004 to 2010, although the incidence of dementia was similar during the same period (Liu et al., 2019). A high incidence of this disease in the older-adult population has a great impact on patients, their family, and the entire society.

The long-term care (LTC) system was set up in Taiwan in 2007 as a response to an aging society. To help families of patients with dementia further, the government in Taiwan established the Long-Term Care (LTC) Act 2.0 in 2017, which covered people with dementia over the age of 50 (Hsu and Chen, 2019). According to the payment categorization in LTC Plan 2.0, long-term care resources were divided into four parts: personal and professional care (including in-home and community-based services such as home personal care or daycare services, home nursing or home rehabilitation services, etc.), transportation services (shuttling from home to hospital), assistive devices (walkers, wheelchairs, commode chairs, relief air mattress, etc.) and in-house barrier-free environment modification (suitable devices that are suggested by therapists and granted partly by the government), and respite care for family caregivers (provided by personal care attendants to allow family caregivers rest for hours to days) (Hsu and Chen, 2019; Yang et al., 2020; Ministry of Health and Welfare, 2022). The goal of LTC Plan 2.0 is to keep aging in place so as to establish an accessible, affordable, and universal long-term care service system. Care management center in the health bureau of each county draws up care plans and links services. Most of the costs are borne by the government (covering by taxes).

Dementia and dependency are predictive factors for LTC service usage and the utilization is related to the level of disability (Wu et al., 2014; Fabius et al., 2022). Older adults' service use patterns partially explain the transitions between community and institutionalization (Chen and Berkowitz, 2012). However, the decision to move to a nursing home or stay resident in the community is complex and multifactorial. Neuropsychiatric symptoms, walking capability, and living status also contribute to the decision of either nursing home stay or home care assistance (Chen et al., 2022). Use of home care assistance service was postulated to potentially lower the probability of nursing home placement (Greiner et al., 2014).

In Taiwan, the overall LTC service utilization rate for patients with dementia was about one-third and home personal care service and assistive devices were the items commonly applied for (Wang W. F. et al., 2021). Drivers of long-term care considerations may be even more complicated. Factors such as dementia stage, capability of activity daily living (ADL), resource of informal and paid care, relationship between the care provider and the recipient, sociocultural context, and the LTC system design all needed to be considered (Morrisby et al., 2018; Shepherd-Banigan et al., 2021). A national dementia registry enrolled 1,268 Taiwanese and revealed that aberrant motor behaviors, dysfunction in ADLs, higher caregiver burden, not residing with family members, and not employing a migrant caregiver were the factors associated with LTC service usage (Tsai et al., 2022).

Investigating dozens of factors at the same time is sometimes effortful and the interpretations become difficult. However, the Apriori algorithm reveals important statistical correlations from viewpoints involving several dimensions or aspects (Chen et al., 2019, 2021; Yan et al., 2019; Lin et al., 2020). Each attribute is viewed as a dimension by appointing threshold values of support and confidence (Han and Kamber, 2006). Therefore, the aim of the present study was to apply the Apriori algorithm to identify the combination of patients with dementia and their caregivers' characteristics associated with LTC services use, especially home personal care service and assistive device in particular.

## Patients and method

This study included 1,201 patients who were diagnosed with mild cognitive impairment or dementia at the memory clinic of Changhua Christian Hospital from October 2015 to June 2021. The diagnosis of dementia was based on the clinical dementia rating (CDR) scale by clinical psychologists (Morris, 1997). Based on the collected data, the use of long-term care services was low among patients with very mild dementia (CDR = 0.5). In order to draw up rules on the usage of LTC services by caregivers with stronger demands, this study excluded the data from patients with very mild dementia. In contrast, this study focused on patients with mild, moderate, and severe dementia, and a total of 475 valid data sets were finally used. The clinical trial number was CCH IRB 220225, which was approved by the Institutional Review Board of Changhua Christian Hospital. In addition, this study used the retrospective study design, while the need for informed consent was waived off by the Institutional Review Board of Changhua Christian Hospital. All data were recorded in the electronic medical chart with the highest confidentiality and compliance with the Declaration of Helsinki.

The variables from patients with dementia consisting of age, gender, marital status, living status, type of dementia, CDR, walking ability, psychological symptoms, mood symptoms, and behavioral symptoms are summarized in Table 1. The care recipient's mood and behavioral and psychological symptoms of dementia (BPSD) for the patients were assessed by using a two-point scale to record the data (1 if the mood/symptom was applied; 0 if not). The presence of BPSD was evaluated by psychologists or trained nursing case managers. Most of the BPSDs listed in the neuropsychiatric inventory (NPI) were recorded by clinical psychologists (Cummings et al., 1994). Other abnormal behaviors, such as pathological crying or laughing, akathisia, wandering, cursing others, and akinesia which were frequently observed in patients with dementia, were also noted by trained nursing case managers. Emotional liability refers to emotional and uncontrollable emotions, and/or emotions that are out of proportion to circumstances, defined by the Diagnostic and Statistical Manual of Mental Disorders, Fifth Edition (DSM-5) (American Psychiatric Association, 2013).

**Table 1:** Demographic information of the patients and data types.

**Variables**		**Frequency**	**Percentage**	**Data type**
Age of the care recipient	< 65 years old	34	7.2	1
	65–74 years old	74	15.6	2
	75–84 years old	260	54.7	3
	≥85 years old	107	22.5	4
Gender of the care recipient	Female	315	66.3	1
	Male	160	33.7	2
Patients' marital status	Married	255	53.7	1
	Divorce	3	0.6	2
	Widow/widower	212	44.6	3
	Separate	0	0	4
	Cohabitation	0	0	5
	Single	5	1.1	6
	Unknown	0	0	7
Patients' living status	Alone	30	6.3	0
	Live with others	437	92.0	1
	Residential care	8	1.7	2
Type of dementia	Alzheimer's disease	271	57.1	1
	Vascular dementia	112	23.6	2
	Mixed dementia	20	4.2	3
	Dementia with Lewy bodies	15	3.2	4
	Parkinson's disease	23	4.8	5
	Alcoholic dementia	0	0	6
	Frontotemporal degeneration	11	2.3	7
	Others	23	4.8	8
CDR of the care recipient	Mild dementia	243	51.2	1
	Moderate dementia	160	33.7	2
	Severe dementia	72	15.1	3
Walking ability of the care recipient	Independent	281	59.2	0
	Walker or cane	144	30.3	1
	Wheelchair	48	10.1	2
	Bedridden	2	0.4	3
Psychological symptoms (multiple choice)	Delusion	113	23.8	1: with the symptom; and 0: without the symptom
	Hallucination	76	16.0	
	Misidentification	35	7.4	
Mood symptoms (multiple choice)	Pathological crying or laughing	22	4.6	
	Phobia	1	0.2	
	Dysthymia	98	20.6	
	Depression	72	15.2	
	Anxiety	39	8.2	
	Worry	24	5.1	
	Anger	97	20.4	
	Irritability	51	10.7	
	Emotional liability	53	11.2	
	Apathy	22	4.6	
Behavioral symptoms (multiple choice)	Agitation	20	4.2	
	Akathisia	9	1.9	
	Wandering	7	1.5	
	Screaming	0	0	
	Curse	14	3.0	
	Shadowing	4	0.8	
	Aggression (verbal/body)	24	5.1	
	Disinhibition	4	0.8	
	Akinesia	26	5.5	
	Nighttime behavior	35	7.4	
	Aberrant motor behavior (stereotype)	10	2.1	

CDR, clinical dementia rating.

Mood and behavioral symptoms were determined by clinical psychologists through neuropsychiatric inventory (NPI). Symptoms outside NPI were evaluated by trained nursing case managers with a binary (yes/no) affirmative response from caregivers: pathological crying/laughing: frequent, brief, intense paroxysms of uncontrollable crying and/or laughing; phobia: fear, dislike, aversion; dysthymia: low grade depressive symptoms; worry: unhappy, frightened; anger: annoyance, displeasure, or hostility; emotional liability: rapid, exaggerated changes in mood; akathisia: restlessness, inability to sit still; wandering: roams around and confused about their location; curse: using more foul language; shadowing: follows someone around like a shadow; akinesia: decreased ability to move voluntarily.

The variables from caregivers including the age, relation to the patient with dementia, employment, marital status, education, type of primary care, frequency of care, caregiver's mood, and Zarit burden interview (ZBI) caregiving burden are summarized in Table 2. The caregiver's mood was assessed by the 12-item Chinese Health Questionnaire (CHQ-12), which is a short, self-administered screening tool for general mental health (Chong and Wilkinson, 1989). Emotional liability included in this study was selected, if rapid, exaggerated changes in the caregiver's mood were noted. The caregiver's mood was assessed by using a two-point scale to record the data, i.e., 1 if the mood was applied; 0 if not. Besides, the ZBI caregiving burden assessed by the Zarit burden interview (ZBI) (Bédard et al., 2001) had four categories, including little or no burden (0–20 points), mild to moderate burden (21–40 points), moderate to severe burden (41–60 points), and severe burden (61–88 points).

**Table 2:** Demographic information of the caregivers and data types.

**Variables**		**Frequency**	**Percentage**	**Data type**
Age of the caregiver	< 50 years old	94	19.8	1
	50–59 years old	183	38.5	2
	60–69 years old	107	22.5	3
	≥70 years old	91	19.2	4
Relation to the VCI patient	Himself/herself	0	0	0
	Spouse	132	27.8	1
	Partner	0	0	2
	Child	274	57.7	3
	Brothers/sisters	3	0.6	4
	Other relatives	65	13.7	5
	Male friends or neighbors	0	0	6
	Female friends or neighbors	0	0	7
	Male foreign worker or household	0	0	8
	Female foreign worker or household	1	0.2	9
Employment	Unemployed or retired	242	50.9	0
	Employed	233	49.1	1
Caregiver's marital status	Married	391	82.3	1
	Divorce	8	1.7	2
	Widow/widower	6	1.3	3
	Separate	0	0	4
	Cohabitation	0	0	5
	Single	68	14.3	6
	Unknown	2	0.4	7
Caregiver's education	Elementary school or below (0–6 years)	78	16.4	1
	Junior high school (7–9 years)	49	10.3	2
	Senior high school (10–12 years)	149	31.4	3
	College and above (≥13 years)	199	41.9	4
Type of primary care	Sole caregiver	165	34.7	1
	Shared caregiving by a caregiver and a foreign worker/household	161	33.9	2
	Shared caregiving by different relatives	11	2.3	3
	Caregiving by a foreign worker	93	19.6	4
	Other	45	9.5	5
Frequency of care	1–2 days per week	36	7.6	1
	3–5 days per week	49	10.3	2
	≥6 days per week	390	82.1	3
Caregiver's mood (multiple choice)	Helplessness	76	16.0	1: with the mood; and 0: without the mood
	Loneliness	20	4.2	
	Depression	35	7.4	
	Anxiety	59	12.4	
	Frustration	52	10.9	
	Nervousness	125	26.3	
	Anger	115	24.2	
	Sadness	23	4.8	
	Emotional liability	51	10.7	
	Troublesome	133	28.0	
	Hopelessness	99	20.8	
ZBI caregiving burden	Little or no burden	136	28.6	1
	Mild to moderate burden	216	45.5	2
	Moderate to severe burden	99	20.8	3
	Severe burden	24	5.1	4

ZBI, Zarit burden interview.

Fifteen long-term care services for older patients with dementia are depicted in Table 3. If patients and their caregivers used a particular LTC service, a value of 1 is assigned. If not, a value of 0 is given. Based on Table 3, assistive devices (27.8%), home personal care services (19.2%), and community care centers (13.1%) were the top three LTC services utilized by patients with dementia and their caregivers.

**Table 3:** Long-term care (LTC) services for older patients with dementia.

**Variables**		**Frequency**	**Percentage**	**Data type**
Long-term care services	Home personal care services	91	19.2	1: with the care service; and 0: without the care service
	Respite care	30	6.3	
	Home nursing care	22	4.6	
	Community care centers	62	13.1	
	Home rehabilitation	23	4.8	
	Assistive devices	132	27.8	
	Adult day care	53	11.2	
	Resettlement	18	3.8	
	Adult foster care	3	0.6	
	Home meal delivery	18	3.8	
	Transportation services	48	10.1	
	Mobile shower	6	1.3	
	Support care for caregivers	17	3.6	
	Barrier-free environment	27	5.7	
	School of wisdom	3	0.6	

The purpose of this study was to identify which attributes from patients and/or caregivers could result in different usages of the LTC services. The Apriori algorithm has been proven to be very effective in identifying statistical correlations from a multidimensional viewpoint in dementia-related areas by setting up support, confidence, and lift (Jhang et al., 2019, 2020; Chang et al., 2021). Thus, the Apriori algorithm was used in this study. The definitions and formulas of support, confidence, and lift are given below (Chang et al., 2021; Jhang et al., 2021). The support of an association rule A ⇒ B is defined by calculating the percentage of transactions containing both A and B in the database depicted in Equation (1):


(1) $$\begin{array}{l} \text{Support }\left(A\Rightarrow B\right)=P\left(A\cap B\right)\\ \;=\frac{\text{number of transactions containing both A and B}}{\text{total number of transactions}}. \end{array}$$

The confidence of the association rule A ⇒ B is used to compute the percentage of transactions containing A and also containing B simultaneously in the database in Equation (2):


(2) $$\begin{array}{l} \text{Confidence }\left(A\Rightarrow B\right)=P\left(B|A\right)=\frac{P\left(A\cap B\right)}{P\left(A\right)}\\ \;=\frac{\text{number of transactions containing both A and B}}{\text{number of transactions containing A}}. \end{array}$$

Lift measures the correlation between A and B, as shown in Equation (3). If a lift has a value of 1, A and B are independent and no rule will be generated. If a lift has a value >1, A and B are dependent and correlated positively.


(3) $$\begin{array}{l} \text{Lift }\left(\text{A},\text{B}\right)=\frac{P\left(A\cup B\right)}{P\left(A\right)P\left(B\right)}. \end{array}$$

The Apriori algorithm in IBM SPSS Modeler 18 was employed. Data type for each variable was defined by the numerical values as shown in Tables 1, 2 for patients with dementia and their caregivers, respectively. The input variables for antecedents from Table 1 were care recipient's age, gender, marital status, living status, type of dementia, CDR, walking ability, three types of psychological symptoms, 10 types of mood symptoms, and 11 types of behavioral symptoms. In addition, the caregiver's age, relation to the patient, employment, marital status, education, type of primary care, frequency of care, 11 types of caregiver's moods, and ZBI caregiving burden from Table 2 were input variables for antecedents. On the other hand, 15 types of LTC services were the input variables for the consequent. Due to the heterogeneous data, the minimum support was set to 2%, whereas the minimum confidence was set to 80% with a lift >1.

## Results

The major characteristics of patients with dementia depicted in Table 1 were female (66.3%), aged 75–84 years (54.7%), and who had Alzheimer's disease (57.1%) with mild dementia (51.2%). In addition, nearly 60% of the patients with dementia could walk independently. On the other hand, 38.5% of the caregivers were aged 50–59 years, and nearly 60% of the caregivers were the children of the patients based on Table 2. More than 80% of the caregivers were married, and 50.9% of the caregivers were unemployed or retired. The type of primary care for care recipient was either sole caregiver (34.7%) or shared caregiving by a caregiver and a foreign worker/household (33.9%). Frequency of care ≥6 days per week was the majority (82.1%). Additionally, the caregiving burden mainly fell into either mild to moderate burden (45.5%) or little or no burden (28.6%).

There were 75 rules generated by the Apriori algorithm with support of 2%, confidence of 80%, and lift >1. Among these rules, 25 belonged to home personal care services and 50 belonged to assistive devices. Based on these 25 rules, four general rules were summarized, based on their similarities to home personal care services. On the other hand, based on these 50 rules, 21 general rules were summarized, based on their similarities to assistive devices.

The first general rule depicted in Table 4 indicated that home personal care services would be required when a sole caregiver whose age was ≥70 years with a mood of helplessness took care of a patient with dementia, regardless of a mix of patient's living status, patient's marital status, caregiver's marital status, frequency of care, or relation to the patient. The second general rule showed that when a sole caregiver who had a mood of helplessness took care of a patient with dementia who had the walking ability by a walker or cane, home personal care services would be needed. The variables of frequency of care and patient's living status were not the critical determinants for home personal care services. For the third general rule, home personal care services would be needed when a sole caregiver who had moods of hopelessness, nervousness, and troublesome took care of a patient with dementia, regardless of the frequency of care and patient's living status. Finally, the fourth general rule indicated that when a caregiver was unemployed or retired and had a mood of hopelessness but who needed to take care of a patient with dementia with a mood symptom of emotional liability, home personal care services should be provided. In addition, frequency of care was not a critical variable to determine the use of home personal care services.

**Table 4:** Four general rules for home personal care services.

**General rule no**.	**Antecedent**	**No. of the cases in the database**	**Support (%)**	**Confidence (%)**	**Lift**
1	Type of primary care: Sole caregiver	10	2.11	80.00	4.18
	Age of the caregiver: ≥70 years old				
	Caregiver's mood: Helplessness				
	Patients' living status: Live with others	10	2.11	80.00	4.18
	Type of primary care: Sole caregiver				
	Age of the caregiver: ≥70 years old				
	Caregiver's mood: Helplessness				
	Patients' marital status: Married	10	2.11	80.00	4.18
	Type of primary care: Sole caregiver				
	Age of the caregiver: ≥70 years old				
	Caregiver's mood: Helplessness				
	Patients' marital status: Married	10	2.11	80.00	4.18
	Type of primary care: Sole caregiver				
	Caregivers' marital status: Married				
	Age of the caregiver: ≥70 years old				
	Caregiver's mood: Helplessness				
	Patients' marital status: Married	10	2.11	80.00	4.18
	Type of primary care: Sole caregiver				
	Age of the caregiver: ≥70 years old				
	Frequency of care: ≥6 days per week				
	Caregiver's mood: Helplessness				
	Patients' living status: Live with others	10	2.11	80.00	4.18
	Patients' marital status: Married				
	Type of primary care: Sole caregiver				
	Age of the caregiver: ≥70 years old				
	Caregiver's mood: Helplessness				
	Type of primary care: Sole caregiver	10	2.11	80.00	4.18
	Caregivers' marital status: Married				
	Age of the caregiver: ≥70 years old				
	Caregiver's mood: Helplessness				
	Type of primary care: Sole caregiver	10	2.11	80.00	4.18
	Caregivers' marital status: Married				
	Age of the caregiver: ≥70 years old				
	Frequency of care: ≥6 days per week				
	Caregiver's mood: Helplessness				
	Patients' living status: Live with others	10	2.11	80.00	4.18
	Type of primary care: Sole caregiver				
	Caregivers' marital status: Married				
	Age of the caregiver: ≥70 years old				
	Caregiver's mood: Helplessness				
	Type of primary care: Sole caregiver	10	2.11	80.00	4.18
	Age of the caregiver: ≥70 years old				
	Frequency of care: ≥6 days per week				
	Caregiver's mood: Helplessness				
	Patients' living status: Live with others	10	2.11	80.00	4.18
	Type of primary care: Sole caregiver				
	Age of the caregiver: ≥70 years old				
	Frequency of care: ≥6 days per week				
	Caregiver's mood: Helplessness				
	Relation to the patient: Spouse	10	2.11	80.00	4.18
	Type of primary care: Sole caregiver				
	Age of the caregiver: ≥70 years old				
	Caregiver's mood: Helplessness				
	Relation to the patient: Spouse	10	2.11	80.00	4.18
	Caregivers' marital status: Married				
	Type of primary care: Sole caregiver				
	Age of the caregiver: ≥70 years old				
	Caregiver's mood: Helplessness				
	Patients' living status: Live with others	10	2.11	80.00	4.18
	Relation to the patient: Spouse				
	Type of primary care: Sole caregiver				
	Age of the caregiver: ≥70 years old				
	Caregiver's mood: Helplessness				
2	Walking ability of the care recipient: Walker or cane	11	2.32	81.82	4.27
	Type of primary care: Sole caregiver				
	Caregiver's mood: Helplessness				
	Walking ability of the care recipient: Walker or cane	11	2.32	81.82	4.27
	Type of primary care: Sole caregiver				
	Frequency of care: ≥6 days per week				
	Caregiver's mood: Helplessness				
	Patients' living status: Live with others	11	2.32	81.82	4.27
	Walking ability of the care recipient: Walker or cane				
	Type of primary care: Sole caregiver				
	Frequency of care: ≥6 days per week				
	Caregiver's mood: Helplessness				
	Patients' living status: Live with others	11	2.32	81.82	4.27
	Walking ability of the care recipient: Walker or cane				
	Type of primary care: Sole caregiver				
	Caregiver's mood: Helplessness				
3	Type of primary care: Sole caregiver	12	2.53	83.33	4.35
	Caregiver's mood: Hopelessness, Nervousness, and Troublesome				
	Type of primary care: Sole caregiver	12	2.53	83.33	4.35
	Frequency of care: ≥6 days per week				
	Caregiver's mood: Hopelessness, Nervousness, and Troublesome				
	Patients' living status: Live with others	12	2.53	83.33	4.35
	Type of primary care: Sole caregiver				
	Frequency of care: ≥6 days per week				
	Caregiver's mood: Hopelessness, Nervousness, and Troublesome				
4	Mood symptoms of the care recipient: Emotional liability	10	2.11	80.00	4.18
	Caregiver's employment: Unemployed or retired				
	Caregiver's mood: Hopelessness				
	Mood symptoms of the care recipient: Emotional liability	10	2.11	80.00	4.18
	Caregiver's employment: Unemployed or retired				
	Frequency of care: ≥6 days per week				
	Caregiver's mood: Hopelessness				

As shown in Table 5, assistive devices were needed when a patient with dementia needed a wheelchair and was cared for by a foreign worker with a frequency of ≥6 days per week (first general rule); when a patient with dementia whose age was 75–84 years had a psychological symptom of hallucination along with a caregiver's mood of troublesome (second general rule); when a female patient with moderate dementia was cared for by a foreign worker (third general rule); when an employed person who had mild to moderate burden needed to provide care for a patient with dementia cared for by a foreign worker (fourth general rule); when a patient with moderate dementia whose age was ≥85 years was cared for by an unemployed or retired caregiver whose age was 60–69 years with mild to moderate caregiving burden (fifth general rule); when a female patient with severe dementia living with others was cared for by a caregiver who had mild to moderate caregiving burden (sixth general rule); when an employed person whose age was 50–59 years with the education of college and above needed to provide care for a patient with dementia cared for by a foreign worker (seventh general rule); when an employed person whose age was 50–59 years provided care for a patient with dementia but whose marital status was widow or widower cared for by a foreign worker (eighth general rule); and when a child provided care for a married patient with Alzheimer's disease cared for by a foreign worker (ninth general rule).

**Table 5:** Twenty-one general rules for assistive devices.

**General rule no**.	**Antecedent**	**No. of the cases in the database**	**Support (%)**	**Confidence (%)**	**Lift**
1	Walking ability of the care recipient: Wheelchair	15	3.16	80.00	2.88
	Type of primary care: Caregiving by a foreign worker				
	Frequency of care: ≥6 days per week				
	Walking ability of the care recipient: Wheelchair	15	3.16	80.00	2.88
	Caregivers' marital status: Married				
	Type of primary care: Caregiving by a foreign worker				
	Frequency of care: ≥6 days per week				
	Patients' living status: Live with others	15	3.16	80.00	2.88
	Walking ability of the care recipient: Wheelchair				
	Type of primary care: Caregiving by a foreign worker				
	Frequency of care: ≥6 days per week				
	Patients' living status: Live with others	15	3.16	80.00	2.88
	Walking ability of the care recipient: Wheelchair				
	Caregivers' marital status: Married				
	Type of primary care: Caregiving by a foreign worker				
	Frequency of care: ≥6 days per week				
	Age of the care recipient: 75–84 years old	11	2.32	81.82	2.94
	Walking ability of the care recipient: Wheelchair				
	Type of primary care: Caregiving by a foreign worker				
	Frequency of care: ≥6 days per week				
	Age of the care recipient: 75–84 years old	11	2.32	81.82	2.94
	Walking ability of the care recipient: Wheelchair				
	Caregivers' marital status: Married				
	Type of primary care: Caregiving by a foreign worker				
	Frequency of care: ≥6 days per week				
	Age of the care recipient: 75–84 years old	11	2.32	81.82	2.94
	Patients' living status: Live with others				
	Walking ability of the care recipient: Wheelchair				
	Type of primary care: Caregiving by a foreign worker				
	Frequency of care: ≥6 days per week				
2	Age of the care recipient: 75–84 years old	11	2.32	81.82	2.94
	Psychological symptoms of the care recipient: Hallucination				
	Caregiver's mood: Troublesome				
	Age of the care recipient: 75–84 years old	11	2.32	81.82	2.94
	Patients' living status: Live with others				
	Psychological symptoms of the care recipient: Hallucination				
	Caregiver's mood: Troublesome				
3	Gender of the care recipient: Female	11	2.32	81.82	2.94
	CDR of the care recipient: Moderate dementia				
	Type of primary care: Caregiving by a foreign worker				
	Gender of the care recipient: Female	11	2.32	81.82	2.94
	Patients' living status: Live with others				
	CDR of the care recipient: Moderate dementia				
	Type of primary care: Caregiving by a foreign worker				
4	Age of the caregiver: 50–59 years old	11	2.32	81.82	2.94
	Caregiver's employment: Employed				
	Type of primary care: Caregiving by a foreign worker				
	ZBI caregiving burden: Mild to moderate burden				
	Patients' living status: Live with others	11	2.32	81.82	2.94
	Age of the caregiver: 50–59 years old				
	Caregiver's employment: Employed				
	Type of primary care: Caregiving by a foreign worker				
	ZBI caregiving burden: Mild to moderate burden				
	Age of the caregiver: 50–59 years old	10	2.11	80.00	2.88
	Caregiver's employment: Employed				
	Type of primary care: Caregiving by a foreign worker				
	Frequency of care: ≥6 days per week				
	ZBI caregiving burden: Mild to moderate burden				
5	Age of the care recipient: ≥85 years old	11	2.32	81.82	2.94
	CDR of the care recipient: Moderate dementia				
	Age of the caregiver: 60–69 years old				
	Caregiver's employment: Unemployed or retired				
	ZBI caregiving burden: Mild to moderate burden				
	Age of the care recipient: ≥85 years old	11	2.32	81.82	2.94
	CDR of the care recipient: Moderate dementia				
	Relation to the patient: Child				
	Caregiver's employment: Unemployed or retired				
	ZBI caregiving burden: Mild to moderate burden				
6	Gender of the care recipient: Female	10	2.11	80.00	2.88
	Patients' living status: Live with others				
	CDR of the care recipient: Severe dementia				
	ZBI caregiving burden: Mild to moderate burden				
	Gender of the care recipient: Female	10	2.11	80.00	2.88
	Patients' living status: Live with others				
	CDR of the care recipient: Severe dementia				
	Caregivers' marital status: Married				
	ZBI caregiving burden: Mild to moderate burden				
7	Age of the caregiver: 50–59 years old	10	2.11	80.00	2.88
	Caregiver's employment: Employed				
	Caregiver's education: College and above				
	Type of primary care: Caregiving by a foreign worker				
	Age of the caregiver: 50–59 years old	10	2.11	80.00	2.88
	Caregiver's employment: Employed				
	Caregivers' marital status: Married				
	Caregiver's education: College and above				
	Type of primary care: Caregiving by a foreign worker				
	Patients' living status: Live with others	10	2.11	80.00	2.88
	Age of the caregiver: 50–59 years old				
	Caregiver's employment: Employed				
	Caregiver's education: College and above				
	Type of primary care: Caregiving by a foreign worker				
8	Patients' marital status: Widow/widower	11	2.32	81.82	2.94
	Age of the caregiver: 50–59 years old				
	Caregiver's employment: Employed				
	Type of primary care: Caregiving by a foreign worker				
	Patients' marital status: Widow/widower	11	2.32	81.82	2.94
	Patients' living status: Live with others				
	Age of the caregiver: 50–59 years old				
	Caregiver's employment: Employed				
	Type of primary care: Caregiving by a foreign worker				
	Age of the care recipient: 75–84 years old	10	2.11	80.00	2.88
	Patients' marital status: Widow/widower				
	Age of the caregiver: 50–59 years old				
	Caregiver's employment: Employed				
	Type of primary care: Caregiving by a foreign worker				
	Gender of the care recipient: Male	10	2.11	80.00	2.88
	Patients' marital status: Widow/widower				
	Age of the caregiver: 50–59 years old				
	Caregiver's employment: Employed				
	Type of primary care: Caregiving by a foreign worker				
	Patients' marital status: Widow/widower	10	2.11	80.00	2.88
	Age of the caregiver: 50–59 years old				
	Caregiver's employment: Employed				
	Caregivers' marital status: Married				
	Type of primary care: Caregiving by a foreign worker				
9	Patients' marital status: Married	15	3.16	80.00	2.88
	Type of dementia: Alzheimer's disease				
	Relation to the patient: Child				
	Type of primary care: Caregiving by a foreign worker				
	Patients' marital status: Married	10	2.11	80.00	2.88
	Type of dementia: Alzheimer's disease				
	Relation to the patient: Child				
	Caregiver's employment: Unemployed or retired				
	Type of primary care: Caregiving by a foreign worker				
10	Age of the care recipient: 75–84 years old	10	2.11	80.00	2.88
	Gender of the care recipient: Female				
	Walking ability of the care recipient: Walker or cane				
	Caregivers' mood: Hopelessness				
	Age of the care recipient: 75–84 years old	10	2.11	80.00	2.88
	Gender of the care recipient: Female				
	Walking ability of the care recipient: Walker or cane				
	Frequency of care: ≥6 days per week				
	Caregivers' mood: Hopelessness				
	Age of the care recipient: 75–84 years old	10	2.11	80.00	2.88
	Gender of the care recipient: Female				
	Patients' living status: Live with others				
	Walking ability of the care recipient: Walker or cane				
	Caregivers' mood: Hopelessness				
11	Patients' marital status: Widow/widower	11	2.32	81.82	2.94
	CDR of the care recipient: Moderate dementia				
	Walking ability of the care recipient: Walker or cane				
	Caregivers' mood: Troublesome				
	Patients' marital status: Widow/widower	10	2.11	80.00	2.88
	Patients' living status: Live with others				
	CDR of the care recipient: Moderate dementia				
	Walking ability of the care recipient: Walker or cane				
	Caregivers' mood: Troublesome				
12	Gender of the care recipient: Female	10	2.11	80.00	2.88
	Patients' marital status: Married				
	Walking ability of the care recipient: Walker or cane				
	Caregiver's education: Senior high school				
	Gender of the care recipient: Female	10	2.11	80.00	2.88
	Patients' marital status: Married				
	Walking ability of the care recipient: Walker or cane				
	Caregiver's education: Senior high school				
	Frequency of care: ≥6 days per week				
	Gender of the care recipient: Female	10	2.11	80.00	2.88
	Patients' marital status: Married				
	Patients' living status: Live with others				
	Walking ability of the care recipient: Walker or cane				
	Caregiver's education: Senior high school				
	Gender of the care recipient: Female	10	2.11	80.00	2.88
	Patients' marital status: Married				
	Walking ability of the care recipient: Walker or cane				
	Caregivers' marital status: Married				
	Caregiver's education: Senior high school				
13	Patients' marital status: Widow/widower	10	2.11	80.00	2.88
	CDR of the care recipient: Moderate dementia				
	Age of the caregiver: 50–59 years old				
	Relation to the patient: Child				
	Caregivers' mood: Troublesome				
	Patients' marital status: Widow/widower	10	2.11	80.00	2.88
	CDR of the care recipient: Moderate dementia				
	Age of the caregiver: 50–59 years old				
	Relation to the patient: Child				
	Frequency of care: ≥6 days per week				
	Caregivers' mood: Troublesome				
14	Age of the care recipient: 75–84 years old	10	2.11	80.00	2.88
	Patients' marital status: Widow/widower				
	CDR of the care recipient: Moderate dementia				
	Frequency of care: ≥6 days per week				
	Caregivers' mood: Troublesome				
	Age of the care recipient: 75–84 years old	10	2.11	80.00	2.88
	Patients' marital status: Widow/widower				
	CDR of the care recipient: Moderate dementia				
	Age of the caregiver: 50–59 years old				
	Frequency of care: ≥6 days per week				
	Caregivers' mood: Troublesome				
15	Age of the care recipient: 75–84 years old	11	2.32	81.82	2.94
	Relation to the patient: Child				
	Frequency of care: ≥6 days per week				
	ZBI caregiving burden: Mild to moderate burden				
	Caregivers' mood: Hopelessness				
	Patients' marital status: Married	11	2.32	81.82	2.94
	Relation to the patient: Child				
	Age of the caregiver: 50–59 years old				
	Frequency of care: ≥6 days per week				
	Caregivers' mood: Hopelessness				
16	Age of the care recipient: 75–84 years old	10	2.11	80.00	2.88
	Walking ability of the care recipient: Walker or cane				
	Caregivers' mood: Troublesome				
	ZBI caregiving burden: Mild to moderate burden				
	Age of the care recipient: 75–84 years old	10	2.11	80.00	2.88
	Gender of the care recipient: Female				
	Relation to the patient: Child				
	Walking ability of the care recipient: Walker or cane				
	Caregivers' mood: Troublesome				
17	Patients' living status: Live with others	11	2.32	81.82	2.94
	Walking ability of the care recipient: Wheelchair				
	Age of the caregiver: 50–59 years old				
	Caregiver's education: College and above				
18	Gender of the care recipient: Female	10	2.11	80.00	2.88
	Patients' marital status: Married				
	Walking ability of the care recipient: Walker or cane				
	Caregivers' marital status: Married				
	Type of primary care: Shared caregiving by a caregiver and a foreign worker/household				
19	Type of dementia: Alzheimer's disease	10	2.11	80.00	2.88
	Relation to the patient: Child				
	Type of primary care: Caregiving by a foreign worker				
	Frequency of care: ≥6 days per week				
	ZBI caregiving burden: Mild to moderate burden				
20	Gender of the care recipient: Male	10	2.11	80.00	2.88
	Type of dementia: Alzheimer's disease				
	Caregiver's employment: Unemployed or retired				
	Caregivers' mood: Troublesome and hopelessness				
21	Mood symptoms of the care recipient: Anger	10	2.11	80.00	2.88
	Walking ability of the care recipient: Walker or cane				
	Caregivers' marital status: Married				
	Caregiver's employment: Unemployed or retired				
	Frequency of care: ≥6 days per week				

Moreover, assistive devices were needed when a female patient with dementia whose age was 75–84 years with the walking ability by a walker or cane was cared for by a caregiver whose mood was hopelessness (tenth general rule); when a patient with moderate dementia whose marital status was widow or widower with the walking ability by a walker or cane was cared for by a caregiver whose mood was troublesome (eleventh general rule); when a female and married patient with dementia who had the walking ability by a walker or cane was cared for by a caregiver whose education was senior high school (twelfth general rule); when a patient with moderate dementia whose marital status was widow or widower was cared for by his or her child whose age was 50–59 years with the mood of troublesome (thirteenth general rule); when a patient with moderate dementia with the age of 75–84 years whose marital status was widow or widower was cared for by a caregiver whose mood was troublesome by a frequency of ≥6 days per week (fourteenth general rule); when a patient with dementia whose age was 75–84 years was cared for by his or her child who had mild to moderate caregiving burden and the mood of hopelessness with the frequency of ≥6 days per week (fifteenth general rule); and when a patient with dementia with the age of 75–84 years who had the walking ability by a walker or cane was cared for by a caregiver who had mild to moderate burden with the mood of troublesome (sixteenth general rule).

From seventeenth to twenty-first general rules as shown in Table 5, each general rule only consisted of one rule due to its rule dissimilarities. When a patient with dementia living with others who needed a wheelchair was cared for by a caregiver whose age was 50–59 years with the education of college and above, assistive devices were required (seventeenth general rule). A female and married patient with dementia who had the walking ability by a walker or cane was cared for by a married caregiver and a foreign worker/household needed assistive devices (eighteenth general rule). When a child who had mild to moderate burden provided care for a patient with Alzheimer's disease was cared for by a foreign worker with the frequency of ≥6 days per week, assistive devices were needed (nineteenth general rule). Assistive devices were required when a male patient with Alzheimer's disease was cared for by an unemployed or retired caregiver who had the moods of troublesome and hopelessness (twentieth). Finally, twenty-first general rule indicated that assistive devices were needed when a patient with dementia with the mood symptom of anger who had the walking ability by a walker or cane was cared for by a married and unemployed or retired caregiver with the frequency of ≥6 days per week.

## Discussion

This study identified certain combinations of the patient–caregiver dyads which resort to either home personal care services or assistive devices for patients with dementia in Taiwan.

In general, patient's living status, patient's walking ability, and mood condition with caregivers' age, caregiving frequency, and employment status were associated with the use of home personal care services. Patients who were taken care of by informal caregivers with the frequency of ≥6 days per week and the caregivers who experienced helplessness or hopeless mood were associated with the use of home personal care services. Patients with emotional liability or being taken care of by their spouses who were aged ≥70 years or unemployed caregivers were also associated with the use of home personal care services. On the other hand, patient's age, marital status, dementia severity, and ambulatory status as well as caregivers' age, type of primary care, frequency of care, caregivers' mood, and caregivers' burden were associated with the use of assistive devices. People living with dementia applying assistive devices were older (≥75 years old), had moderate to severe dementia, needed assistance in walking or were wheelchair bound, and were being cared for mainly by a foreign care helper.

As in Western countries, LTC service is a complex package in Taiwan that is allocated to use different services in the same manner regarding the severity of disability and dementia (Feldman et al., 2021; Van Cleve and Degenholtz, 2022). Our findings provided a glimpse of the LTC service usage among patient–caregiver dyads of patients with dementia in Taiwan. Generosity and accessibility of the LTC service also contribute to the results (Wysocki et al., 2015; Wang S. et al., 2021).

Factors that are related to institutionalization and assisted living service in community sometimes overlapped. Physical function, cognition, and mood condition are worthy of keeping an eye on. Not surprisingly, the health profiles including age, dementia severity, and walking ability are associated with the LTC service usage (Liu et al., 2018). Care recipient's dependency is positively correlated with the utilization of LTC services (Feldman et al., 2021; Van Cleve and Degenholtz, 2022). This profile may drive the LTC service to fit their users better.

Caregivers' employment status also appears to be in relation to the association rules. Feldman et al. (2021) also found caregivers not working had a greater likelihood of service usage. Behavioral and psychological symptoms presented in 80% of the dementia population from the onset of cognitive dysfunction lead to heavy burden on both the patients and their caregivers (Lyketsos et al., 2002; Baharudin et al., 2019). Home health aides and home personal care services are reported to reduce caregivers' burden in mean caregiver strain scores (Reckrey et al., 2021). Patients with emotional liability, anxiety mood, and hallucination are found to be associated with the LTC usage in the present study. Our study also reveals that the picture of higher strain of caregiving presenting as a feeling of being over relied on may force the patients and their caregivers to resort to both services. It is worth considering the need of social services, physical function, cognition, and mood condition. Mood conditions of both patients and their caregivers are worth recording and intervention. Non-pharmacological and pharmacological treatments for BPSD, as well as the caregiver support group and education to relieve caregiver's helplessness, are important assistance measures for caregivers who take care of people with dementia (Livingston et al., 2005; McLoughlin, 2022).

The strength of the present study includes comprehensive characteristics of people living with dementia and their caregivers, as well as care mode and care load. Another strength of the present study is the fact that through using the Apriori algorithm, the caring scenario associated with the LTC service usage could be elucidated. However, there are some study limitations. First, when applying the Apriori algorithm, there is no universal approach to set up support and confidence values in order to generate association rules. In general, a higher confidence value, say 90% or above, is recommended when a conditional probability is applied to study the associations of attributes. In contrast to confidence, setting a support value is usually correlated with the diversity of factors included in the analysis. Higher support values reduce the number of rules and easily summarize meaningful results, whereas lower support values help in generating some essential rules with low frequencies. Because of the heterogeneity between patients' and caregivers' characteristics and the usage of LTC services, the confidence and support values were set as 80% and 2% in order to generate association rules. No rules could achieve above 90% confidence value, which weakened the conditional probability of associations. Second, some important factors associated with LTC service usage, including average family income and accessibility of the LTC service, were not included in the present study. Third, the cross-sectional design made it difficult to determine the causal relationship between the correlates and the usage of LTC services.

This study provides preliminary results on the LTC service usage from people living with dementia and their caregivers residing in the community. Understanding the patient–caregiver dyad's profile leads the service providers, policymakers, and the referral team to tailor service provisions better to meet the needs and identify the potential target groups.

## Conclusion

This study identified 75 rules on the usage of LTC services by a comprehensive analysis of the characteristics of patients with dementia and their caregivers. There were 25 rules belonging to home personal care services, which could be summarized further into four general rules. Patient's walking ability (by a walker or cane), patient's emotional liability, unemployed or retired caregivers, caregivers' feelings with either helplessness or hopelessness, and caregivers who cared for the patients with dementia solely were found to be the critical variables to use home personal care services. On the contrary, patient's walking ability (mainly by a walker or cane), age, and severity as well as caregivers' age, mood, marital status, caregiving burden, and the patient being cared for mainly by a foreign care helper were found to be the critical variables to use assistive devices services. Aside from each patient's and caregiver's basic characteristics and caregiving load, the present study highlighted that mood conditions of both patients and caregivers were associated with LTC services usage. It is worthy to check and give an appropriate intervention for the abnormal mood status of people with dementia and their caregivers. These general rules serve as references to provide either home personal care services or assistive devices to reduce caregivers' burden as well as to improve the quality of care for patients with dementia.

## Data availability statement

The datasets presented in this article are not readily available because following our hospital's regulation, individual de-identified data should pass both the Research and Development Committee and Institutional Review Board (IRB). Researchers should write a research proposal and apply a clinical trial for the IRB of our hospital. All data listed in the present manuscript can be obtained through email if both committees agree the application. Further inquiries can be directed to the corresponding author.

## Ethics statement

The Clinical Trial Number was CCH IRB 220225, which was approved by the Institutional Review Board of Changhua Christian Hospital. Written informed consent for participation was not required for this study in accordance with the national legislation and the institutional requirements.

## Author contributions

All authors listed have made a substantial, direct, and intellectual contribution to the work and approved it for publication.
